# Medical Codes, Etc.

**Published:** 1883-07-20

**Authors:** 


### *MEDICAL CODES, ETC.*

A great deal is now being said in relation to the Code of Ethics. That there is a growing disregard to the observance of the time-honored code of medical ethics, is plain to every observing man—a fact which we would fain believe is not attributable to any increased indifference to correct and honorable principles, or to any growing demoralization in the profession at large. It is due rather to the evasion of the Code by indirect methods on the part of many prominent teachers and specialists throughout the country, who advertise themselves by their writings circulated among the masses, and by procuring publicity through the public press of their remarkable operations.

To what extent this mode of advertising may be legitimately done, as in the plan of circulating reprints of medical articles from the journals, has been a subject of dispute, it being claimed that the writer who has the intelligence and the energy to get up a readable and practical article, is to be commended for his enterprise and is legitimately entitled to all the honor and benefits which may accrue to him from his labors in the field of medical progress and medical literature.

We admit the force and plausibility of this argument. Yet it is true that this, or the abuse of it is one of the factors which has weakened the respect and the hold which the Code has, in times past, exercised upon the men of moderate attainments and of ordinary position in the ranks of the profession.

Another and powerful factor, which is telling upon the Code is the utter ignorance of the non-professional public of the Science of Medicine—their want of appreciation of its great benefits to mankind, and of its need of protection and encouragement as against imposters and mountebanks; and of that universal license to all forms of quackery which seems to be incident to our free government and liberal institutions.

Statesmen and patriots of conservative views are constantly exclaiming against the reckless and dangerous latitudinarianism which may result to society from an over-free and unbridled democracy. The same dangers exist in respect to the learned professions, not excluding theology and the profession of medicine.

As to the remedy for these evils, there is little to hope from mere written codes or from legal enactments. Laws are of no avail as against a wide-spread and antagonistic public sentiment. We can only look to increased educational and moral development of the whole people. And in this the medical journalism of the country holds an important and highly responsible position. Equally and perhaps more important to the promotion of this great reform is the work of our medical colleges, —a higher order of medical education, not only in respect to medical training, but especially in respect to honor, to truth and to the highest moral rectitude.

In this great work which can only be conducted by the slow yet persistent and persevering inculcation of correct principles, it is essentially important that the non-professional public should, in some way, be reached, and herein is the greatest difficulty to be encountered.

Now that the American Medical Association is to have a special organ for the publication of its transactions, can there not be a supplemental or separate department gotten out for the non-professional reader, designed and conducted with the view of educating the masses in regard to the great evils of quackery, to the importance of sustaining legitimate medicine, and to the obligations of the public to the medical profession? Of course it could not, at once, be hoped to extend these instructions to the masses, but it would perhaps not be impracticable to reach many of the more intelligent and influential class of readers, especially the members of the legal profession and the clergy who, by their testimonials to patent nostrums, and their patronage to all forms of charlatanism, have done more to disparage legitimate medicine and to sustain quackery than all other classes combined. W.

